# Comparison of the amyloid pore forming properties of rat and human Alzheimer’s beta-amyloid peptide 1-42: Calcium imaging data

**DOI:** 10.1016/j.dib.2016.01.019

**Published:** 2016-01-16

**Authors:** Coralie Di Scala, Nouara Yahi, Alessandra Flores, Sonia Boutemeur, Nazim Kourdougli, Henri Chahinian, Jacques Fantini

**Affiliations:** a Aix-Marseille Université, PPSN-EA4674, Faculté des Sciences, Marseille, France; a INSERM, INMED, Parc Scientifique de Luminy, 13009 Marseille, France

## Abstract

The data here consists of calcium imaging of human neuroblastoma SH-SY5Y cells treated with the calcium-sensitive dye Fluo-4AM and then incubated with nanomolar concentrations of either human or rat Alzheimer’s β-amyloid peptide Aβ1-42. These data are both of a qualitative (fluorescence micrographs) and semi-quantitative nature (estimation of intracellular calcium concentrations of cells probed by Aβ1-42 peptides vs. control untreated cells). Since rat Aβ1-42 differs from its human counterpart at only three amino acid positions, this comparative study is a good assessment of the specificity of the amyloid pore forming assay. The interpretation of this dataset is presented in the accompanying study “Broad neutralization of calcium-permeable amyloid pore channels with a chimeric Alzheimer/Parkinson peptide targeting brain gangliosides” [1].

**Specifications Table**

**Table:** 

Subject area	*Neurosciences*
More specific subject area	*Molecular basis of neurodegenerative diseases*
Type of data	*Text file and figures*
How data was acquired	*Fluorescent microscope*
Data format	*Analyzed*
Experimental factors	*Cells treated with the calcium-sensitive dye Fluo-4AM and then incubated with either human or rat A*β*1-42 peptide.*
Experimental features	*Control cells are preloaded with Fluo-4AM and then treated with buffer alone to determine background intracellular calcium levels. This background is subtracted from the values induced by either human or rat A*β*1-42.*
Data source location	*Not applicable*
Data accessibility	*All data are presented in this article*

**Value of the data**

• The data provides a comparative study of Ca^2+^ fluxes induced by human and rat forms of Aβ1-42 peptide.
• The comparison of the Ca^2+^ fluxes induced by human and rat Aβ1-42 peptides may be used as an internal reference to validate this assay for studying amyloid pore formation induced by any amyloid protein in living neural cells.
• Researchers interested in testing amyloid pore formation and inhibitors of amyloid pores might carefully choose positive and negative controls with calibrated compounds.

## 1 Data

**Fig. 1:**
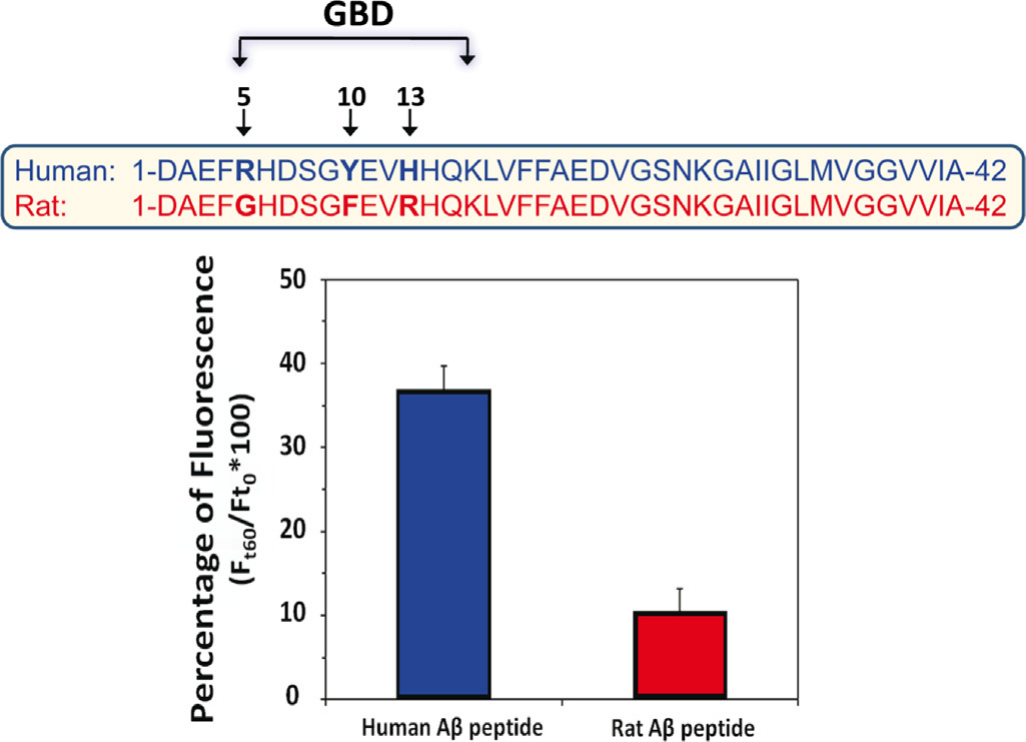
Ca^2+^ fluxes induced by human and rat Aβ1-42 peptides in SH-SY5Y cells: a comparative study. Amino acid sequence alignments (upper panel) show that human and rat Aβ1-42 peptides differ at only three positions, all located in the ganglioside-binding domain (GBD) [1], [12]. SH-SY5Y cells treated with the calcium dye Fluo-4AM were incubated with human Aβ1-42 (blue histogram, *n*=87) or rat Aβ1-42 (red histogram, *n*=120) and calcium-dependent fluorescence was recorded after 60 min of incubation with the indicated peptides (lower panel). Results are expressed as mean±SEM. Statistical significance between human and rat Aβ1-42 (*p*=2.75×10^−11^) was evaluated by using Student׳s *t*-test.

**Fig. 2:**
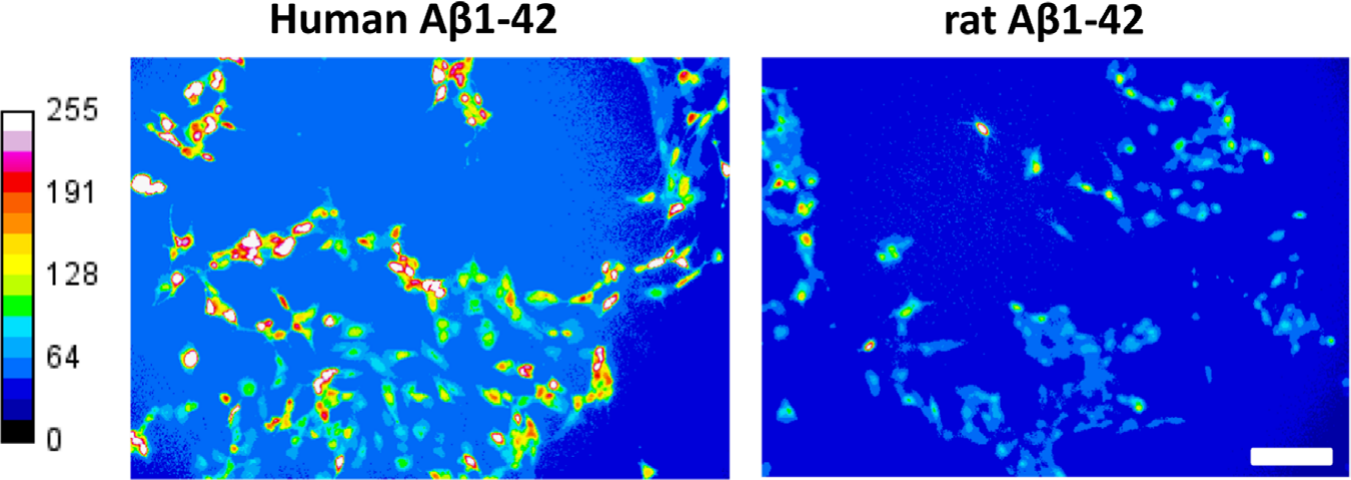
Cell imaging of Ca^2+^ fluxes induced by human and rat Aβ1-42 peptides in SH-SY5Y cells: a comparative study. The images show pseudocolor representations of cells (scale bar: 100 µm), warmer colors corresponding to higher fluorescence. The photographs are taken after 60 min of incubation with each peptide (same experimental conditions as in Fig. 1).

Amyloid pores [1], [2], [3], [4] are responsible for a dramatic increase of intracellular Ca^2+^ levels in brain cells that can be measured by fluorescence microscopic imaging [5], [6], [7], [8], [9], [10], [11], [12]. The dataset presented here contains the values of intracellular Ca^2+^ concentrations induced by human and rat Aβ1-42 peptides in neural SH-SY5Y cells. Amino acid sequence alignments of these peptides are presented in Fig. 1. Quantitative data (histograms) are given in Fig. 1, and fluorescence micrographs are shown in Fig. 2.

## 2 Experimental design, materials and methods

### 2.1 Materials

SH-SY5Y cells were obtained from ATCC. Dulbecco׳s Modified Eagle Medium: Nutrient Mixture F12 (DMEM/F12), HBSS, glutamine and penicillin/streptomycin were furnished by Gibco. Fluo-4AM was from Invitrogen. Human and rat Aβ1-42 peptides were purchased from rPeptide. The purity of these peptides was >95% as assessed by HPLC.

### 2.2 Cell culture

SH-SY5Y cells were cultured in DMEM/F12 supplemented with 10% fetal calf serum, glutamine (2 mM) and penicillin (50 U/mL)/streptomycin (50 µg/mL) and maintained at 37 °C with 5% CO_2_. Cells were passaged twice a week and not used beyond passage 25.

### 2.3 Calcium measurements

SH-SY5Y cells were plated (45.000 cells/dish) in 35 mm culture dishes and grown during 72 h at 37 °C. They were loaded with 5 µM Fluo-4AM for 30 min in the dark as previously described [1], [5]. The calcium fluxes were estimated by measuring the variation of cell fluorescence intensity after the injection of either rat or human Aβ1-42 (220 nM) into the recording chamber directly above an upright microscope objective (BX51W Olympus) equipped with an illuminator system MT20 module. Fluorescence emission at 525 nm was imaged by a digital camera CDD (Hamanatsu ORCA-ER) after fluorescence excitation at 490 nm. Time-lapse images (1 frame/10 s) were collected using the CellR Software (Olympus). Fluorescence intensity were measured from region of interest (ROI) centered on individual cells. Signals were expressed as fluorescence after treatment (*F*_t60_) divided by the fluorescence before treatment (*F*_0_) and multiplied by 100. The results were averaged and the fluorescence of control is subtracted of each value. The experiments were performed at 30 °C during 60 min. In the pseudocolor representations of cells, warmer colors correspond to higher fluorescence and thus to higher Ca^2+^ levels.

## 3 Statistical analysis

Quantitative data are expressed as mean±S.E.M. and the statistical significance was assessed with the Student׳s *t*-test.
